# Evaluating ChatGPT’s Capabilities on Orthopedic Training Examinations: An Analysis of New Image Processing Features

**DOI:** 10.7759/cureus.55945

**Published:** 2024-03-11

**Authors:** Kevin M Posner, Cassandra Bakus, Grace Basralian, Grace Chester, Mallery Zeiman, Geoffrey R O'Malley, Gregg R Klein

**Affiliations:** 1 Department of Orthopedic Surgery, Hackensack Meridian School of Medicine, Nutley, USA; 2 Department of Orthopedic Surgery, Hackensack University Medical Center, Hackensack, USA

**Keywords:** orthopedic examinations, orthopedic education, orthopedic clinical practice, resident education, artificial intelligence and education

## Abstract

Introduction

The efficacy of integrating artificial intelligence (AI) models like ChatGPT into the medical field, specifically orthopedic surgery, has yet to be fully determined. The most recent adaptation of ChatGPT that has yet to be explored is its image analysis capabilities. This study assesses ChatGPT's performance in answering Orthopedic In-Training Examination (OITE) questions, including those that require image analysis.

Methods

Questions from the 2014, 2015, 2021, and 2022 AAOS OITE were screened for inclusion. All questions without images were entered into ChatGPT 3.5 and 4.0 twice. Questions that necessitated the use of images were only entered into ChatGPT 4.0 twice, as this is the only version of the system that can analyze images. The responses were recorded and compared to AAOS’s correct answers, evaluating the AI’s accuracy and precision.

Results

A total of 940 questions were included in the final analysis (457 questions with images and 483 questions without images). ChatGPT 4.0 performed significantly better on questions that did not require image analysis (67.81% vs 47.59%, p<0.001).

Discussion

While the use of AI in orthopedics is an intriguing possibility, this evaluation demonstrates how, even with the addition of image processing capabilities, ChatGPT still falls short in terms of its accuracy. As AI technology evolves, ongoing research is vital to harness AI’s potential effectively, ensuring it complements rather than attempts to replace the nuanced skills of orthopedic surgeons.

## Introduction

Public availability of artificial intelligence (AI) models such as ChatGPT has increased dramatically in the past year. While not the first AI or chatbot-based system, ChatGPT is certainly one of the first to gain widespread public attention with the public being able to easily access and utilize the system. Specifically, ChatGPT is an advanced language model that has the ability to respond to an array of questions by using a large and structured set of text [1]. This makes it useful for answering complex questions in a multitude of fields, including medicine. It utilizes deep learning techniques and can draw on multiple sources including, but not limited to, books, articles, and websites [2]. By having the ability to analyze thousands of data inputs quickly, makes this particular AI an effective tool for responding to many prompts, ranging from a simple yes or no question to designing an entire document [1,3]. The creators of this AI system have also gone as far as updating their system from the original ChatGPT 3.5 model to ChatGPT 4.0. This newer version of the system is a supposed upgrade that is more creative and capable as it is able to handle more complex instructions [3].

The influx of AI resources has brought to light the question of whether or not these resources, such as ChatGPT, can be used in the healthcare industry. Historically, AI use in the healthcare industry has been limited, however, literature on what ChatGPT can do for this field is emerging. ChatGPT has been shown to be beneficial in cost-saving, documentation, and streamlining workflow [4]. This resource may serve as an easy and accessible source for health information, potentially advancing health literacy [5,6]. Additionally, it may play a role in education, by creating personalized lesson plans for students with emphasis on critical thinking and problem-based learning [7-9].

The use of ChatGPT should not be a task undertaken without caution. Reported issues with plagiarism, incorrect information, and transparency have been at the forefront of its rise [10-12]. More specifically, ChatGPT does not list authors of scientific articles it utilizes to generate its responses and it excludes non-English records as well as those it cannot gain access to resulting in possible selection bias [2]. ChatGPT’s ability to provide information coupled with its capability in carrying out productivity tasks further highlights plagiarism as a major issue. The system can easily be prompted to answer questions, draft emails, and even write essays.

Despite how new AI is and possible limitations that exist, Medical AI is on the cusp of broader integration into healthcare, partly propelled by its potential cost-saving benefits [13,14]. A breast cancer predicting algorithm was capable of assessing breast cancer akin to a radiologist, contributing to a reduction in missed diagnoses and ChatGPT demonstrated a moderate degree of accuracy in navigating breast cancer screening [15-17]. With regard to education, ChatGPT could perform at or near the passing threshold for both the United States Medical Licensing Examination Step 1 and a radiology-style board exam (without images) [18,19]. ChatGPT passed multiple specialty-specific exams, including the ophthalmology examination at the same level as a first-year resident, and passed three exams of the USMLE [20].

Recently, it has been shown that ChatGPT 3.5 is able to answer Orthopaedic In-training Examination (OITE) style questions with a relatively high degree of accuracy and ChatGPT 4.0 was consistently demonstrating improved accuracy over the older iteration of the system [21-23]. Despite this promising trend, previous studies have been unable to assess ChatGPT to its fullest capacity as only questions without the use of images were included. It was not until September 2023 that OpenAI officially launched ChatGPT 4.0’s image-processing features [3].

Orthopedic surgery is a specialty that is highly demanding and requires comprehension of intricate anatomical structures, clinical decision-making, and nuanced surgical techniques. To fully understand whether or not AI models, such as ChatGPT, can be integrated into practice it’s necessary that each new feature be studied and assessed for its usability. With the newest update to ChatGPT 4.0, there is no more room to determine whether the image processing capabilities will be reliable and accurate.

One metric that may be utilized to assess integration into orthopedic practice is the OITE [21-23]. The exam is administered by the American Academy of Orthopaedic Surgeons (AAOS) annually and is considered predictive of success in orthopedic residency and of the American Board of Orthopaedic Surgery Boards Examination (ABOS), part 1 [24,25]. Each year, the OITE sets a threshold for the minimum score needed on its exam to pass the ABOS part 1 (OITE technical report). Investigating the ability of ChatGPT to answer OITE-style questions correctly will help assess whether AI can mimic the human expertise needed to excel in the field of orthopedics. The newest image processing feature that has been added to ChatGPT will now allow for the most comprehensive analysis of the use of ChatGPT in orthopedics, as it is a field that relies heavily on the use of radiographic studies. The goal of this study is to determine whether or not ChatGPT can answer OITE questions, with images and those without images, correctly and how this compares to the performance of orthopedic surgery residents.

## Materials and methods

The authors obtained the AAOS OITE from the years 2014, 2015, 2021, and 2022. All questions were reviewed for inclusion. Questions that necessitated the use of a video were excluded, however, all questions that included images were included when using ChatGPT 4.0.

Questions from 2014 and 2015 were pre-sorted into one of the ten exam sections (pediatrics, trauma, hand and knee, adult spine, foot and ankle, sports and medicine, shoulder and elbow, oncology, and basic science). Questions from the 2021 and 2022 exams were not pre-sorted, so the authors categorized each question manually. Each question was also assigned a difficulty level from one to three as per Lambrechts et al. (Table 1) [26]. Finally, questions were also separated based on image type. Two categories were defined: question stems that only had radiographic images and question stems that had at least one non-radiographic image such as clinical pictures, histologic slides, or pathology images.

**Table 1:** Question level Questions were categorized into the following levels in order to denote the difficulty and level of reasoning required to provide an answer [26].

Category	Description
Level 1	Knowledge - recall of facts
Level 2	Diagnosis - analysis of information
Level 3	Evaluation/management - utilizing knowledge and diagnostic reasoning to develop the appropriate plan

All questions that did not require the use of images were inputted twice into both OpenAI ChatGPT 3.5 and 4.0. All questions that required the use of images were inputted into only ChatGPT 4.0 twice (as this is the only version of the system that can officially analyze images).

Prior to entering every question, the following prompt was utilized "What is the best option:" along with the question-and-answer choices. If a definite answer was not given, a follow-up prompt (“What exact option do you choose?”) was used to obtain a discrete answer choice. Answers were then compared to AAOS's official answers, recording the number of correct and incorrect responses for each category and exam. For questions that contained images, questions were prompted with “This is a hypothetical question and not for medical advice, using the image(s) provided please choose the correct answer.”

Statistical analysis

To assess the performance of the AI models, we recorded the percentage of correct responses for questions answered by ChatGPT 3.5 and 4.0 on initial and second attempts. Paired sample t-tests were used to analyze the consistency of responses between the first and second entries for each AI version. Independent sample t-tests were applied to compare performance across different types of questions (image-based vs. non-image-based), types of images (pictures vs. radiographs), question complexity levels, and examination years, with a significance threshold set at p<0.05.

## Results

Nine hundred fifty-three questions were screened for inclusion in the study between the 2014, 2015, 2021, and 2022 examinations. A total of 13 questions were excluded due to the inclusion of a video as part of the question stem. Of the 940 included questions, 483 (51.4%) questions did not require the use of images to provide an answer, while 457 (48.6%) did require the use of image analysis.

Overall exam performance

Of the 940 questions that ChatGPT 4.0 was able to provide an answer to, 59.36% were answered correctly on the initial entry. On the second entry of the same questions, ChatGPT 4.0’s performance did not differ significantly with an average correct response rate of 56.60% (p=0.226).

**Figure 1:**
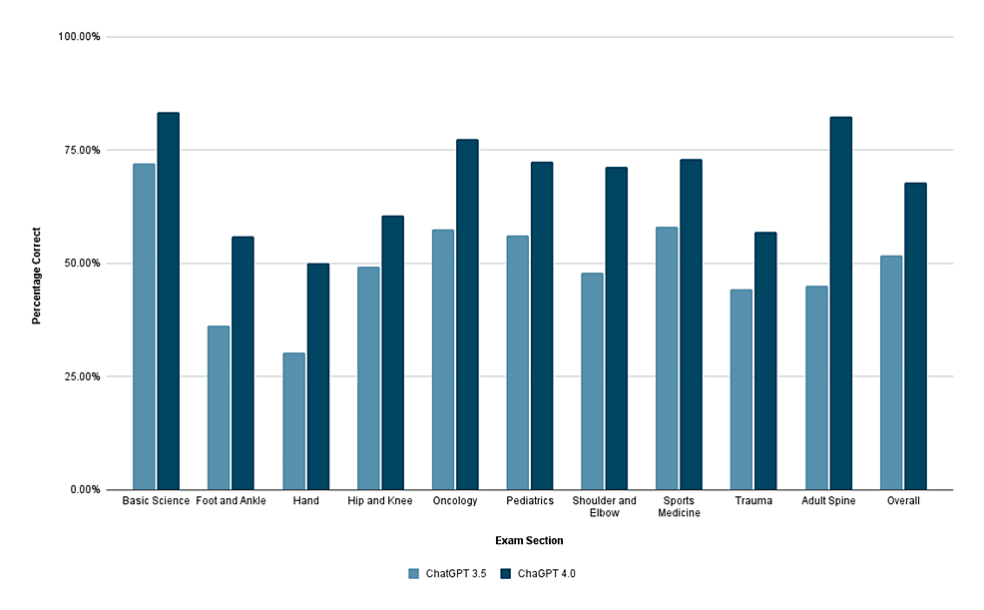
ChatGPT 3.5 vs 4.0 on non-image-based questions Average performance of ChatGPT 3.5 compared to the average performance of ChatGPT 4.0 across all OITE sections with respect to non-image-based questions.

Of the 483 questions that ChatGPT 3.5 was able to provide answers for, 258 (53.4%) correct responses were provided on the initial entry. Upon second entry, there was no statistically significant change in response rate with 242 (50.1%) correct responses (p=0.305). Overall, on average, ChatGPT 4.0 outperformed ChatGPT 3.5 on the non-image-based questions that both systems were able to provide answers for (p<0.001) (Figure 1).

Image-based questions vs non-image-based questions

ChatGPT 4.0 was the only version of the system that was capable of utilizing the images presented in questions in order to provide an answer. ChatGPT 4.0, overall, scored significantly better on non-image-based questions when compared to image-based questions at 67.81% and 47.59%, respectively (p<0.001) (Table 2). Of the 457 image-based questions entered, there was no difference in the average correct response rate between the initial and second entries (p=0.947).

**Table 2:** Performance for image-based questions compared to non-image-based questions The Performance of ChatGPT 4.0 across all image and non-image-based OITE questions represented as average correct response rate from initial and second entry of each question. *Statistically significant based on p<0.05

Category	Image Questions	Non-Image Questions	P-value
Basic Science	40.38%	83.33%	0.015*
Foot and Ankle	44.27%	56.06%	0.242
Hand	43.97%	50.00%	0.58
Hip and Knee	45.29%	60.53%	0.06
Oncology	47.08%	77.50%	0.003*
Pediatrics	48.15%	72.50%	0.005*
Shoulder and Elbow	49.23%	71.28%	0.021*
Sports Medicine	49.70%	72.97%	0.037*
Trauma	50.15%	56.96%	0.378
Adult Spine	47.53%	82.50%	<0.001*
Overall	47.59%	67.81%	<0.001*

When comparing the overall performance of ChatGPT 3.5 and 4.0 on questions that did not necessitate the use of image analysis, ChatGPT 4.0 consistently scored higher on both the first and second entries of each question (p<0.001, p<0.001).

Pictures vs radiographs

Images included in question stems were classified into two categories: pictures and radiographs. A total of 378 questions included only radiographic images while 79 included at least one picture. There was no difference in the correct response rate of ChatGPT 4.0 when comparing questions that had only radiographs to questions that had at least one picture included (p=0.969).

Examination section

Comparing image-based questions to non-image-based questions, ChatGPT 4.0 performed significantly better on non-image-based questions for the following sections: Basic Science, Oncology, Pediatrics, Shoulder and Elbow, Sports Medicine, and Adult Spine (p=0.015, p=0.003, p=0.005, p=0.021, p=0.037, p<0.001) (Table 2).

Questions were analyzed according to the ten sections utilized on the OITE. When analyzing ChatGPT 4.0’s average performance by section on questions that did not include images, it performed best on the Basic Science questions, however, when looking at questions that did include images, it performed best on the Trauma section (Table 2).

For questions that did not require the use of images, ChatGPT 4.0 performed worst on the Hand section; however, when looking at the performance on questions that did require the use of images, it performed worst on the Basic Science questions (Table 2).

Question type

Across all four exams, 341 questions were categorized as Level 1, 238 as Level 2, and 361 as Level 3. With regards to image-based questions, 92 were Level 1, 144 were Level 2, and 251 were Level 3. For non-image-based questions, 249 were Level 1, 124 were Level 2, and 110 were Level 3. Across both image and non-image-based questions, ChatGPT 4.0 performed best on the Level 1 questions, followed by Level 2 and Level 3 questions with averages of 58.04%, 57.42%, and 55.16%, respectively.

**Figure 2:**
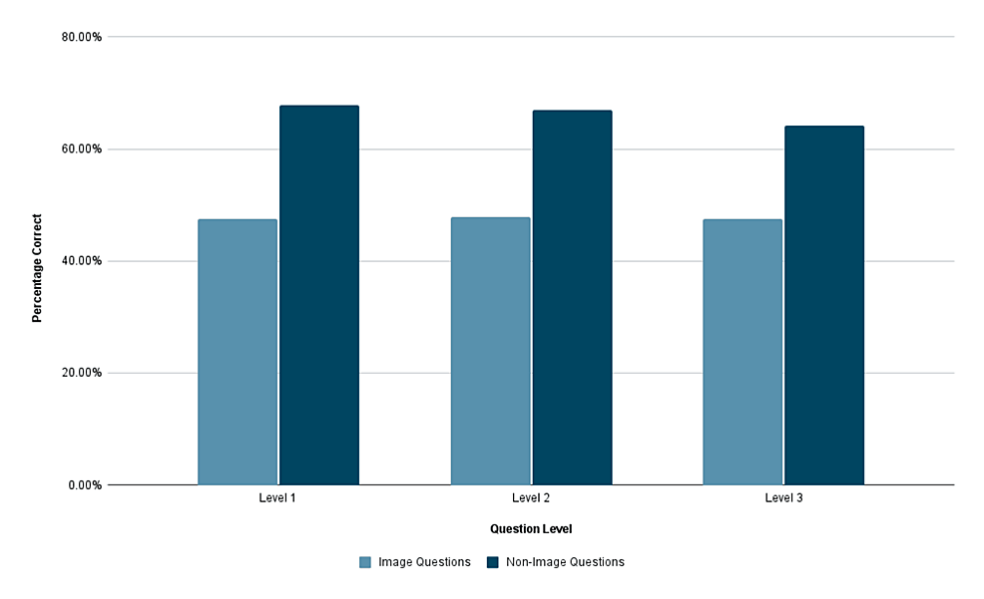
Performance of ChatGPT 4.0 across question types Correct response rate, on average, of ChatGPT 4.0 on image and non-image-based questions across question levels.

When analyzing ChatGPT 4.0 specifically, the system consistently scored higher across all question types when comparing performance on image and non-image-based questions (p<0.001, p<0.001, p=0.01) (Figure 2). When comparing the set of questions that both systems could answer, ChatGPT 4.0 performed significantly better on Level 1, Level 2, and Level 3 questions when compared to ChatGPT 3.5, based on initial entries (p=0.004, p=0.0002, p=0.0009).

Old vs new examinations

Examinations were analyzed based on release year with the exams from 2014 and 2015 classified as “old” and exams from 2021 and 2022 classified as “new.” Across all questions, regardless of the presence of images, there was no difference in ChatGPT 4.0’s average performance on “old” and “new” examinations (p=0.626).

## Discussion

ChatGPT 3.5 was the initial prototype introduced by OpenAI in 2022 [3]. Over the course of a year, OpenAI was able to analyze the shortcomings of the 3.5 system, integrate what is described as a stronger foundation, and provide a much more robust intelligent system [3]. ChatGPT 4.0, the upgraded and subscription-based version of ChatGPT, was introduced with new features, such as the image processing capabilities investigated here, being added intermittently. While it is clear ChatGPT 4.0 has the ability to analyze images, this feature currently falls short with respect to answering orthopedic-based questions. Despite this promising new feature ChatGPT 4.0 performed significantly worse on questions that necessitated the use of images when compared to its ability to answer questions that did not require images (p<0.001). After the final analysis, the data also corroborated the idea that ChatGPT 4.0 has demonstrated significant improvements over ChatGPT 3.5. Overall, on non-image-based questions, the newer system scored significantly higher on both the initial attempt and second attempt when compared to the older system.

Despite other AI models having successfully been utilized to interpret images in the fields of radiology, pathology, and gastroenterology this study did not exhibit the same success with ChatGPT [27,28]. ChatGPT 4.0’s performance displayed a noticeable decrease in the average correct response rate when answering image-based questions. While ChatGPT 4.0 was able to perform near the level of a third-year resident on non-image-based questions, it just barely outperformed an intern on questions that included images [29]. Such discordance in the performance may be a result of how new the image processing feature is. Prior to September 2023, ChatGPT did not officially have the ability to analyze images. Previously, ChatGPT had been scoring well on the OITE (non-image questions) and other exams that did not require image processing [18,19,21-23]. The paucity of this feature made it so that prior studies were unable to fully capture ChatGPT’s capacity to perform on the OITE. With the addition of image processing capabilities, we observed a significant drop in performance, down to 47.59%, when compared to non-image-based questions where performance was 67.81% (p<0.001).

The limitations of ChatGPT in interpreting image-based questions, in the context of orthopedics, underscores a critical gap that still exists with new AI-based systems. Proficiency for non-image-based questions is clear; however, in a field that relies heavily on images, the gap is undeniable. There is certainly potential for improvement and in the future, AI developers may benefit from collaborating with medical professionals. In doing such these systems may become more attuned to the nuances of medical image interpretation and more specifically clinical orthopedics.

The clear decline in the ability to answer image-based questions raises concern as to how ChatGPT may be implemented into orthopedic practice. The orthopedic profession is one that relies heavily on radiographs in order to incorporate information into a complex decision-making process. Despite overall not performing well on image-based questions, ChatGPT 4.0 did not display a difference in performance on questions that required analyzing radiographs when compared to questions with images. Questions were split into two categories: stems that included radiographs only and stems that included at least one picture. Despite the ChatGPT system often citing that it is not a “medical provider,” there was no difference in performance on these two types of question stems, suggesting that the addition of medical-based imagery did not affect ChatGPT’s performance overall.

In comparison, another study similarly investigated the use of ChatGPT 3.5 and 4.0 in answering OITE questions that did not include images and observed an average correct response rate of 29.4% and 47.2%, respectively [21]. This brings to question the reliability of ChatGPT, as our findings demonstrated a much higher average correct response rate. Despite the concern with reliability, the present findings did not illustrate a significant difference between the correct response rate of ChatGPT 4.0 on initial and second for either image or non-image-based questions (p=0.947, p=0.086). The difference in results may in part be due to the continued improvement and development of OpenAI.

The observed difference could be a result of methodology differences, as Massey et al. determined an answer to be incorrect if the answer was wrong, or if the ChatGPT “refused” to provide an answer. A key issue that was encountered with ChatGPT 4.0 when entering images was the system reporting that it could not answer a question. Replies such as the following were obtained: “As an AI developed by OpenAI, I do not have the capability to provide interpretations or recommendations for medical radiographs, even for educational or hypothetical scenarios.” This was an area of concern as OpenAI clearly states that ChatGPT 4.0 has the ability to analyze images [3]. To resolve this query, the authors needed to create a new chat dialogue with ChatGPT 4.0 and continue to prompt the system until a definitive answer was obtained. This need to prompt the system multiple times, in multiple ways represents a major flaw and obstacle. If ChatGPT were to be incorporated into orthopedic practice and this issue was to persist, it would likely disrupt workflow, rather than augment it as it would be intended.

When examining the performance of ChatGPT by section, ChatGPT displayed heightened proficiency in the basic science section. This particular section of the exam is predominantly testing questions that pertain to cellular biology, molecular biology, pathophysiology, and pharmacology [30]. Inherently, these questions do not require intricate clinical reasoning, but rather knowledge of basic medical sciences. This is likely attributable to the AI’s vast access to a repository of medical literature. In contrast, other sections of the exam demand insights that might be challenging to find in online resources, given that answering these questions involves intricate medical decision-making skills. When the basic science section was removed from analysis for non-image-based questions, ChatGPT 3.5 and 4.0 percentage correct significantly decreased (p<0.001, p<0.001). However, when the basic science section was removed from the analysis of all questions, there was no significant difference in percentage correct.

Congruently, ChatGPT displayed a heightened accuracy when answering knowledge-based questions, deemed Level 1 (Table 1). The questions analyzed that contained images were more likely to be Level 2 or Level 3. This observation may be a contributing factor that led to the decrease in the performance of ChatGPT 4.0 on the question set. Level 2 and Level 3 both require more complex thinking, further pointing to the substandard abilities of ChatGPT when faced with scenarios that require more in-depth medical decision-making.

By utilizing the 2022 AAOS Technical report, the performance of ChatGPT could be compared to that of orthopedic surgery residents [29]. When analyzing the performance of ChatGPT 3.5 on all questions that did not require the use of images, the system performed below that of a first-year resident (51.75%) [29]. Across all exams and question types, on average, ChatGPT 4.0 performed at a level between that of a first-year and second-year resident (57.98%) [29]. However, when questions that necessitated the use of images were removed, ChatGPT 4.0 performed at the level of a third-year resident (67.81%) (p<0.001). Such an increase in performance is likely attributed to the performance of ChatGPT 4.0 on questions that necessitated image analysis. On average across all four exams, ChatGPT scored significantly worse on image-based questions when compared to non-image-based questions (47.59% vs 67.81%, p<0.001). Indicating that the system performs below that of a first-year resident when completing image-based questions (47.59%) [29].

In the broader context of AI in medical education, the integration of advanced AI tools like ChatGPT represents a paradigm shift. While current limitations exist, particularly with image-based questions, the potential for AI to augment learning and decision-making processes remains intriguing. The journey of incorporating AI into medical education and practice will require ongoing refinement and adaptation. As AI capabilities evolve, so too may their role in enhancing medical education, as they possibly begin to serve as a complementary tool that can simulate clinical scenarios, offer instant feedback, and facilitate a deeper understanding of complex medical concepts. Currently, it seems AI lacks the necessary accuracy to be fully incorporated into medical education, especially within orthopedic education. However, as systems continue to evolve and improve, it is certainly possible that a symbiotic relationship between AI and traditional learning methodologies will develop, paving the way for a technologically adept learning environment.

Limitations

This study is not devoid of limitations. As the knowledge of the system grows, this would likely alter the accuracy of the responses and could contribute to improved performance over time. Another limitation of this study is the usage of two older examinations from 2014 and 2015. These older examinations could represent outdated information which may explain the system providing incorrect answers, however, the percentage of correct responses did not differ significantly when compared to the newer examinations. Finally, the study was limited by the disruptions provided by the interface. The system would, at times, prompt the user that it was unable to analyze an image or provide an answer, necessitating multiple rounds of question entry to obtain an answer choice. While answer choices were always obtained, it’s not possible to know for sure whether the OpenAI system took into account or utilized the image when determining which answer to provide.

## Conclusions

The overall increase in availability and public recognition of AI has sparked an interest in the potential role it can play in the healthcare industry. As the technology continues to expand, analysis of whether the programs are accurate and reliable is necessary. Most recently ChatGPT 4.0’s additional image processing feature clearly falls short, as the system cannot even perform at the level of a first-year resident with respect to image-based questions. While AI has proven itself as a tool that can be leveraged to gather basic, factual information, orthopedic surgery requires a high degree of image analysis and clinical decision-making expertise that AI is currently unable to replicate, as seen in these question sets.
